# Establishment of the endocrine variant extractor and its clinical application in identifying a novel *GATA3* mutation in HDR syndrome

**DOI:** 10.3389/fendo.2026.1840651

**Published:** 2026-07-01

**Authors:** Yunseo Han, Danbi Song, Minsoo Noh, Mikyung Kang, Young Sil Eom, Hunsang Lee, Sihoon Lee

**Affiliations:** 1 Department of Life Sciences, Korea University, Seoul, Republic of Korea; 2 National Research Laboratory for Convergence Degradation Biology, Korea University, Seoul, Republic of Korea; 3 Division of Endocrinology and Metabolism, Gachon University Gil Medical Center, Incheon, Republic of Korea; 4 Laboratory of Genomics and Translational Medicine, Department of Internal Medicine, Gachon University College of Medicine, Incheon, Republic of Korea

**Keywords:** computational biology, endocrine disease, exome sequencing, genetic variation, parathyroid diseases, sequence analysis, DNA

## Abstract

**Background:**

Genetic diagnosis of endocrine disorders is often hampered by the complexity of analyzing Whole Exome Sequencing (WES) data. We developed the endocrine variant extractor (EVE), a streamlined, clinician-friendly bioinformatics pipeline designed for multi-tier genetic screening with a core panel for parathyroid disorders (26 genes) and an expanded endocrine panel for broader metabolic assessment (413 genes, fully encompassing the parathyroid panel).

**Methods:**

Encapsulated within a Docker container and automated via a custom Python wrapper, EVE integrates core bioinformatics engines, including BWA-MEM, GATK, and SnpEff. The pipeline employs a tiered reporting strategy, filtering and annotating variants across both panels using pathogenicity scores (SIFT, PolyPhen-2) and clinical databases (ClinVar, gnomAD). This architecture ensures cross-platform compatibility without complex manual configuration.

**Results:**

To validate the pipeline, EVE was applied to clinical datasets. EVE successfully filtered >300,000 raw variants down to a handful of actionable candidates. Using this pipeline, we precisely identified the first Korean case of a *de novo GATA3* frameshift variant (p.Ala173fs) in an HDR syndrome patient, which was not previously reported in the ClinVar database. Analysis took ~3 h, reducing manual data review by >99.6%.

**Conclusion:**

EVE provides a streamlined, high-efficiency workflow that automates the filtering of thousands of raw WES variants into a curated list of clinically relevant variants. This robust framework enables the creation of a comprehensive “endocrine variant atlas,” empowering clinicians to integrate high-throughput genetic profiling into routine diagnostics and accelerate the discovery of novel disease-causing variants. The complete source code for EVE is freely available at https://github.com/hanyunseo01/EVE.

## 1 Introduction

Next-generation sequencing (NGS) has revolutionized clinical diagnostics (1), offering unprecedented insight into the molecular basis of endocrine disorders (2). However, the sheer scale of genomic data presents a significant barrier to its routine clinical application. The human genome comprises approximately 3 billion base pairs (3), and whole exome sequencing (WES), which targets only the protein-coding regions, generates massive data. A typical exome analysis may identify between 20,000-100,000 variants per individual, of which most are benign single nucleotide polymorphisms (SNPs) or variants of uncertain significance (4). For the practicing endocrinologist, this creates a “needle in a haystack” dilemma (5). Although WES offers an unbiased and comprehensive look at the genome, filtering thousands of background SNPs to pinpoint the singular pathogenic variant responsible for a patient’s phenotype is challenging. This challenge is further compounded in endocrinology, in which clinical presentations often overlap across different metabolic pathways, necessitating a broader genomic perspective beyond single-organ panels (6). Furthermore, the lack of standardized software environments often leads to reproducibility issues, whereby the same data yields different results depending on the computing infrastructure (7). Without a standardized and accessible bioinformatic workflow, the potential of WES to diagnose the full spectrum of endocrine disorders remains largely untapped in clinical practice. To address these challenges, we present the endocrine variant extractor (EVE), a user-friendly computational pipeline designed for the non-bioinformatician. To ensure strict reproducibility and ease of deployment, the workflow is encapsulated within a Docker container, eliminating dependency conflicts common in genomic analysis (8).

This protocol outlines a step-by-step method starting from unbiased WES data, progressing through alignment and variant calling, and culminating in a two-tiered filtering strategy that analyzes a core parathyroid panel and an expanded endocrine panel. By integrating pathogenicity predictions (SIFT (9), PolyPhen-2 (10)) and clinical databases (ClinVar (11)), EVE facilitates the rapid identification of causal variants, supporting the immediate goal of personalized treatment.

Such a streamlined approach is necessary for rare and heterogeneous conditions like Hypoparathyroidism, deafness, and renal dysplasia (HDR) syndrome, also known as Barakat syndrome (12, 13). HDR syndrome is an autosomal dominant disorder caused by heterozygous loss-of-function mutations in *GATA3* (14), a transcription factor essential for embryonic development of the parathyroid glands, inner ear, and kidneys. Since its first description by Barakat et al. (12), fewer than several hundred cases showing highly variable expression have been reported (15, 16). Many patients do not exhibit the full triad, partly due to the temporal divergence in symptom onset (17), making clinical diagnosis challenging in the absence of genetic confirmation. In this study, we evaluated a 28-year-old woman presented with long-standing hypocalcemia and sensorineural hearing loss, but without renal manifestations. By utilizing the EVE to her WES data, we identified a *de novo* frameshift variant in *GATA3* (c.517delG; p.Ala173fs). This identification provides further insight into the genotype-phenotype correlation of HDR syndrome. Such an integrated approach highlights the diagnostic value of the EVE framework in resolving atypical clinical presentations of idiopathic hypoparathyroidism and establishes a reproducible model for genomic analysis in endocrine practice. Ultimately, our work contributes to the long-term objective of compiling a comprehensive “endocrine variant atlas” to facilitate future genomic diagnostics.

## 2 Materials and methods

### 2.1 Ethical statement

This study was approved by the Institutional Review Board of Gachon University Gil Medical Center (No. GDIRB2023-336). Informed consent was obtained from the patient and her family members for the genetic analysis and the publication of this case report. All studies were conducted in accordance with relevant ethical guidelines and regulations.

### 2.2 Sample collection and DNA preparation

Whole-exome libraries were prepared using the SureSelect XT Human All Exon V5 Kit (Agilent Technologies, USA) according to the manufacturer’s instructions. Briefly, 800 ng of high-quality genomic DNA was sheared into fragments of approximately 150–200 bp using a Covaris S220 ultrasonicator (Covaris, USA). The fragmented DNA was end-repaired, A-tailed, and ligated to Illumina paired-end adapters using the reagents provided in the kit. Adapter-ligated DNA fragments were purified using AMPure XP beads (Beckman Coulter, USA) and amplified by limited-cycle polymerase chain reaction (PCR) to obtain a pre-capture library. The pre-capture libraries were hybridized with SureSelect Human All Exon V5 capture probes for 24 hours at 65 °C to selectively enrich exonic regions. The resulting DNA–RNA hybrids were captured using streptavidin-coated magnetic beads, followed by a series of washes to remove nonspecific bindings. The enriched libraries were quantified using qPCR and assessed for size distribution using an Agilent 2100 Bioanalyzer (Agilent Technologies, USA). Equimolar amounts of libraries were pooled and sequenced on the Illumina HiSeq 2500 platform (Illumina, USA) using 150 bp paired-end reads according to the manufacturer’s standard protocols. Image analysis and base calling were performed using the Illumina CASAVA software. Raw sequencing reads were subjected to adapter trimming and quality filtering before downstream alignment and variant calling.

### 2.3 Study design and data input

**Figure 1:**
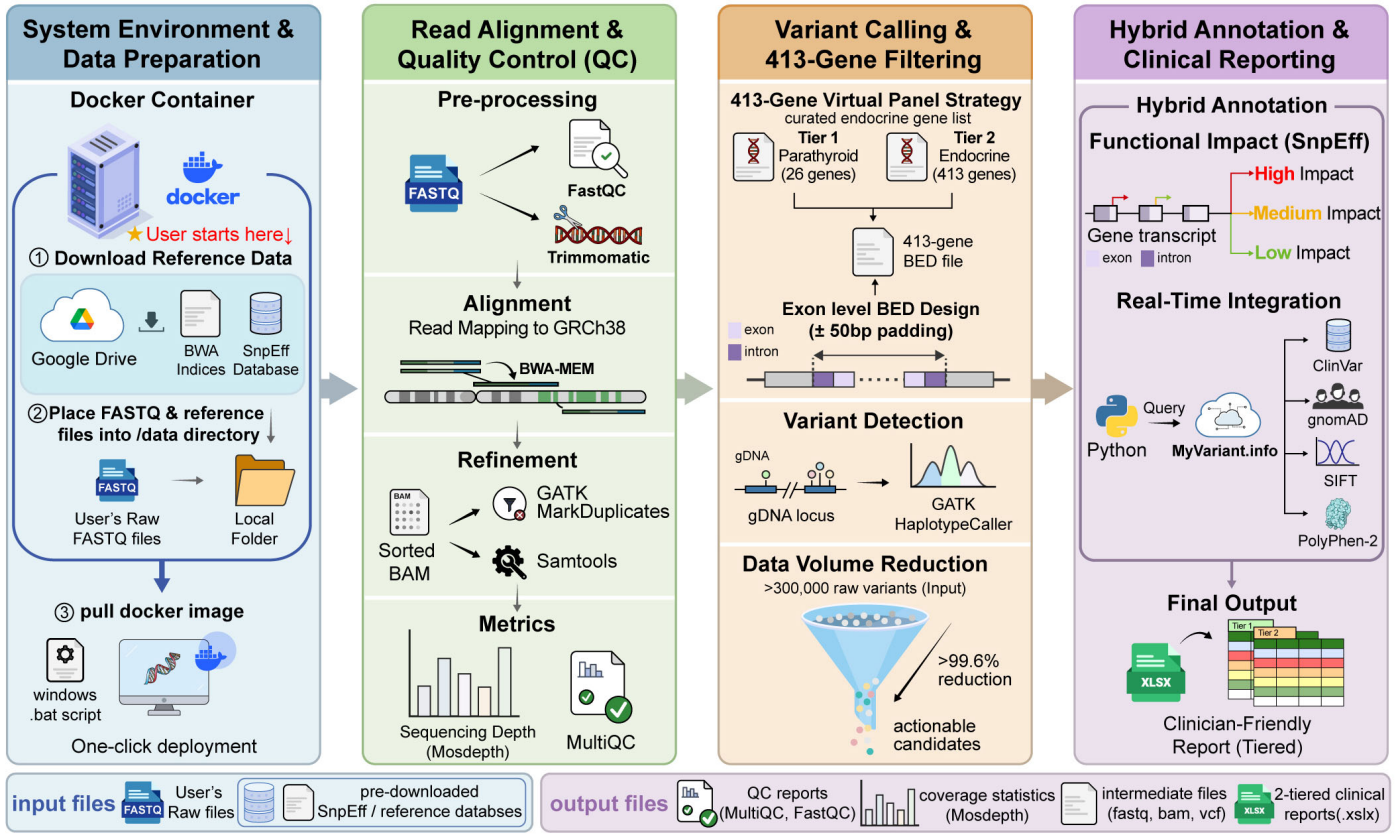
Schematic workflow of the endocrine variant extractor (EVE) pipeline. The process begins with System Environment & Data Preparation, where the workflow is encapsulated in a Docker container to ensure cross-platform accessibility. Heavy reference data, such as BWA indices and SnpEff databases, are externalized to maintain a lightweight image. In the Read Alignment & Quality Control (QC) stage, raw FASTQ files are processed via FastQC and Trimmomatic, aligned using the BWA-MEM algorithm, and refined through GATK MarkDuplicates and Samtools. For Variant Calling & 413-Gene Filtering, GATK HaplotypeCaller is utilized within a 413-gene virtual panel strategy (combining Tier 1 parathyroid and Tier 2 endocrine expansion panels) with ±50bp interval optimization. This targeted approach achieves a >99.6% reduction in data volume, narrowing raw variants down to actionable candidates. Finally, the Hybrid Annotation & Clinical Reporting module uses SnpEff to predict functional impact and a Python script to perform real-time queries of ClinVar, gnomAD, SIFT, and PolyPhen-2 via MyVariant.info, culminating in a tiered Excel report for clinical use.

EVE is orchestrated by a custom Python wrapper script that initiates the automated, sequential execution of bioinformatics tools directly from raw FASTQ input files to the generation of a final tiered clinical report in Excel format (**Figure 1**). The required input for each EVE run is a single patient’s paired-end FASTQ files (R1 and R2). To address large-scale genomic databases distribution, we adopted a “lightweight containerization strategy.” The core analysis environment, including FastQC (18), Trimmomatic (19), GATK (v4.4.0.0) (20), BWA (21), Samtools (22), SnpEff (23), Mosdepth (24) and Python dependencies, was encapsulated within a minimal Docker image. This design creates an isolated, consistent operating environment that eliminates dependency conflicts. Unlike traditional monolithic containers, massive annotation databases and reference genome indices are externalized to reduce image size and ensure pipeline stability. This design bypasses the potential failure of internal automated downloads caused by frequently changing URLs or unstable connectivity of public genomic repositories. Consequently, users are required to download pre-computed BWA indices for GRCh38 and SnpEff databases from the provided repository before initiating the pipeline. Although the containerized architecture inherently supports UNIX-based systems (Linux and macOS), we specifically developed a batch execution script (.bat) for Windows-prevalent clinical settings to enable “one-click” analysis by automatically checking for these downloaded resources and mapping local data directories to the container’s internal file system. For analysis of multiple patients, EVE can be executed in parallel by launching independent Docker containers, with each container mounting a separate patient’s data directory.

### 2.4 Read alignment and quality control

Raw sequencing reads undergo rigorous quality control within the container. We use FastQC to assess read quality and Trimmomatic to remove adapter sequences and low-quality bases. Adapter trimming was performed using the TruSeq3-PE library sequences. Low-quality bases were removed using a sliding window approach (window size; 4 bp, average Phred quality threshold; 15). Additionally, leading and trailing bases with a quality score ≤ 3 were trimmed and reads ≤ 36 base pairs post-trimming were discarded. Following cleanup, paired-end reads are aligned to the pre-computed human reference genome (GRCh38) using the BWA-MEM (25) algorithm with default parameters. Post-alignment, depth of coverage is calculated using Mosdepth to ensure sufficient sequencing depth across target regions. The resulting BAM files are sorted, indexed, and processed for duplicate removal using Samtools and GATK MarkDuplicates to mitigate PCR amplification bias. Finally, MultiQC (26) is employed to aggregate quality metrics from all steps into a single comprehensive report.

### 2.5 Targeted variant calling and the virtual panel filter

Variant calling is performed using the GATK HaplotypeCaller, the industry gold standard for germline variant detection. To streamline the analysis for clinical relevance, EVE implements an automated multi-tier virtual panel filter. Before initiating the variant calling process, the pipeline merges the core parathyroid panel (26 genes (27)) and the endocrine expansion panel (413 genes, fully encompassing the parathyroid panel) into a single unified target BED file (the full list of genes is available in **Supplementary Tables S1**, **S2**). The expanded endocrine gene list was adopted from the panel routinely used for genetic diagnosis at Yonsei University Health System, reflecting clinical experience accumulated from an established endocrine genetics program (28). The target BED file was generated at the exon level using exon coordinates retrieved from the MyGene.info API, with ±50 bp padding flanking each exon to ensure detection of canonical splice-site variants and proximal intronic mutations of clinical relevance (29). This exon-level design maintains high diagnostic sensitivity for clinically actionable variants while significantly reducing overall processing time. EVE also supports user-defined custom panels, allowing users to add or remove genes according to their specific clinical or research focus; detailed instructions are provided in the GitHub repository and the accompanying video tutorial. By passing the unified target interval directly to the GATK interval argument (-L), the analysis is restricted to specific regions of interest. This approach optimizes computational efficiency and minimizes incidental findings by focusing solely on phenotype-relevant candidates.

### 2.6 Hybrid variant annotation and impact prediction

Filtered variants are subsequently processed to assess their clinical and functional significance. The EVE pipeline automates this annotation via a two-step hybrid approach implemented within the Python orchestration script.

First, the pipeline executes SnpEff (v5.1d) with the -*canon* flag to annotate the variant call format (VCF) file against the pre-downloaded GRCh38 SnpEff database, restricting the output to canonical transcripts in order to minimize redundant entries and ensure a single, unambiguous coordinate per gene. SnpEff stores the resulting annotation in a structured ANN field, which the Python script parses at the transcript level to populate the Gene, Effect, Impact, Transcript ID, DNA Change, and Protein Change columns of the report. In parallel, the script constructs HGVS genomic identifiers in the form chrom:g.pos ref>alt for each variant and submits real-time queries to the MyVariant.info web service (29). From this single query, the script retrieves dbSNP rsIDs, ClinVar clinical significance classifications, gnomAD allele frequencies, and dbNSFP pathogenicity scores, which populate the Variant ID, ClinVar, gnomAD AF, SIFT, and PolyPhen columns. Finally, the Chromosome, Position, Ref, and Alt columns are extracted directly from the corresponding VCF columns and retained for secondary validation. The pipeline consolidates all annotated variants into a unified pandas DataFrame and exports them as an Excel report comprising two sheets: a parathyroid panel (26 core genes) and an endocrine expansion panel (413 genes).

### 2.7 Structure of the EVE clinical report

**Figure 2:**
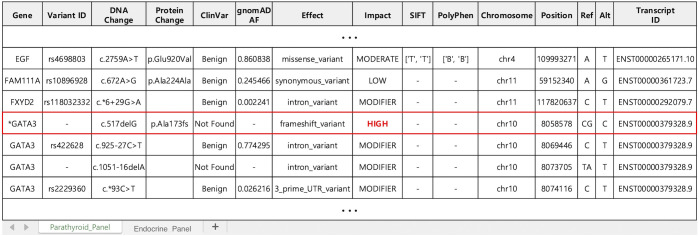
Representative layout of the EVE clinical report illustrating the hybrid variant annotation schema. Each row corresponds to a single variant. Functional annotations (Gene, DNA Change, Protein Change, Effect, Impact, Transcript ID) are parsed from the canonical-transcript SnpEff ANN field (-*canon* flag), while clinical and population-level annotations (Variant ID/rsID, ClinVar, gnomAD AF, SIFT, PolyPhen) are retrieved in real time from the MyVariant.info web service. Chromosome, Position, Ref, and Alt columns are extracted directly from the VCF and reported against the GRCh38 reference assembly. SnpEff Impact tiers: HIGH, predicted loss-of-function (e.g., frameshift, stop-gain); MODERATE, non-synonymous changes (e.g., missense, in-frame indels); LOW, silent changes (e.g., synonymous variants); MODIFIER, non-coding or regulatory variants. SIFT prediction codes: D, Deleterious; T, Tolerated. PolyPhen-2 (HumVar) prediction codes: D, Probably damaging; P, Possibly damaging; B, Benign. Array values (e.g., [‘T’, ‘T’]) indicate predictions across multiple transcript isoforms reported by dbNSFP. The boxed row highlights the *de novo GATA3* frameshift variant (c.517delG; p.Ala173fs) identified as the sole HIGH-impact candidate in the parathyroid panel of the proband.

A representative layout of the EVE clinical report is shown in **Figure 2**. Each row represents a single variant annotated with data derived from the SnpEff ANN field, the MyVariant.info web service, and the original VCF file.

The report includes the HGNC-approved gene symbol and the dbSNP reference identifier (Variant ID). The DNA Change and Protein Change columns denote variants using standard Human Genome Variation Society (HGVS) nomenclature calculated against the canonical Ensembl transcript (Transcript ID). Clinical significance is extracted from the NCBI ClinVar database (11), while global allele frequencies are retrieved from the gnomAD genomes dataset (gnomAD AF).

Functional consequences are described using Sequence Ontology terms (Effect) and categorized into four severity tiers defined by SnpEff (Impact). For missense variants, complementary in silico pathogenicity predictions from SIFT (9) and PolyPhen-2 (10) are provided. Detailed prediction codes and impact tier definitions are summarized in the **Figure 2** legend.

Finally, genomic coordinates (Chromosome, Position, Ref, and Alt) are anchored to the GRCh38 reference assembly. Because the report is generated as a standard Excel file, clinicians can easily filter and sort these variables to apply ACMG/AMP-aligned criteria and prioritize variants tailored to specific diagnostic contexts without requiring bioinformatics expertise.

### 2.8 Validation of the identified mutation by sanger sequencing

Genomic DNA was extracted from peripheral blood samples obtained from the proband and available family members (father, mother, and sister) using the NucleoSpin Tissue Mini Kit for DNA from cells and tissue (Macherey-Nagel, Cat. No. 740952, Germany) according to the manufacturer’s instructions. The genomic region encompassing the identified variant was amplified by polymerase chain reaction (PCR) using gene-specific primers (**Supplementary Table S3**). Each PCR reaction was performed in a 50 μL mixture containing approximately 50 ng of genomic DNA, 2.5 μL of 10 pmol primer solution for each primer, and 25 μL of 2× Phanta Flash Master Mix (Vazyme, Cat No. P520, China). Thermal cycling was performed according to the manufacturer’s instructions. PCR products were verified by agarose gel electrophoresis and subsequently purified using the PURE™ PCR Cleanup Kit (Infusion Tech, Cat. No. PRG411, Republic of Korea). Purified amplicons were submitted to Bionics Korea (Seoul, Korea) for Sanger sequencing. The resulting chromatograms were analyzed and aligned to the reference genome (GRCh38) using the SnapGene software (GSL Biotech, USA) to confirm the presence and familial segregation of the identified mutation.

### 2.9 Code availability and implementation

The EVE container is distributed via Docker Hub (hanyunseo01/eve:v3.0, also accessible via the ‘latest’ tag), ensuring environment consistency across Windows, macOS, and Linux platforms. The heavy reference data and annotation databases are hosted separately; access links to download these essential bundles (via Google Drive) are explicitly provided in the GitHub repository documentation. The complete source code, including the Dockerfile, Python orchestration script, and Windows batch launcher, is openly available in a public repository (https://github.com/hanyunseo01/EVE). This integrated distribution model guarantees that clinicians can deploy a reproducible, research-grade bioinformatics pipeline using a single command, mitigating the technical barriers encountered in routine genomic diagnostics. To further enhance accessibility for clinician users, a step-by-step video tutorial demonstrating the complete EVE workflow is publicly available on YouTube, with a direct link provided on the main page of the GitHub repository.

## 3 Results

### 3.1 Pipeline validation and performance evaluation in clinical settings

To demonstrate the utility of the proposed pipeline, we validated each analysis tier using two distinct clinical datasets: (1) an in-house WES dataset from a patient suspected of HDR syndrome and (2) a publicly available dataset (SRR23072658) from a patient with bilateral adrenocortical adenomas (30). For each case, the total analysis time from raw FASTQ to clinical report was approximately 3 h. Analysis was performed on a Linux-based server environment allocated with 20 GB RAM, using 4 CPU threads for parallel processing of genomic data. To evaluate the filtering efficacy of the EVE pipeline, we compared a standard non-targeted analysis against our targeted approach using the same bioinformatics pipeline. The only procedural difference was the GATK analysis mode: the control group was processed in non-targeted WES mode, while the EVE group utilized a targeted mode focusing on 413 curated gene targets from the Tier 1 and Tier 2 panels. For the in-house dataset, the initial non-targeted variant calling identified 325,957 raw variants. By applying the integrated EVE filtering framework, the candidate pool was drastically reduced to 1,217 variants, representing a 99.63% reduction. Similarly, for the public adrenocortical adenoma dataset, the raw variant count was narrowed from 400,906 to 1,285, effectively excluding over 99.68% of the genomic background noise.

Crucially, when prioritized through our tiered filtering logic, this approach achieves a reduction of over 99.6% in the volume of data requiring manual review for primary diagnostic targets. These results demonstrate that EVE significantly streamlines the diagnostic workflow by excluding hundreds of thousands of irrelevant variants, thereby highlighting the efficiency of the targeted approach for routine clinical diagnostics in complex endocrine pathologies.

### 3.2 Pathogenicity prediction and clinical correlation

To evaluate the diagnostic utility of the pipeline, the findings from the EVE-generated clinical reports were compared with the patients’ observed endocrine pathologies.

**Figure 3:**
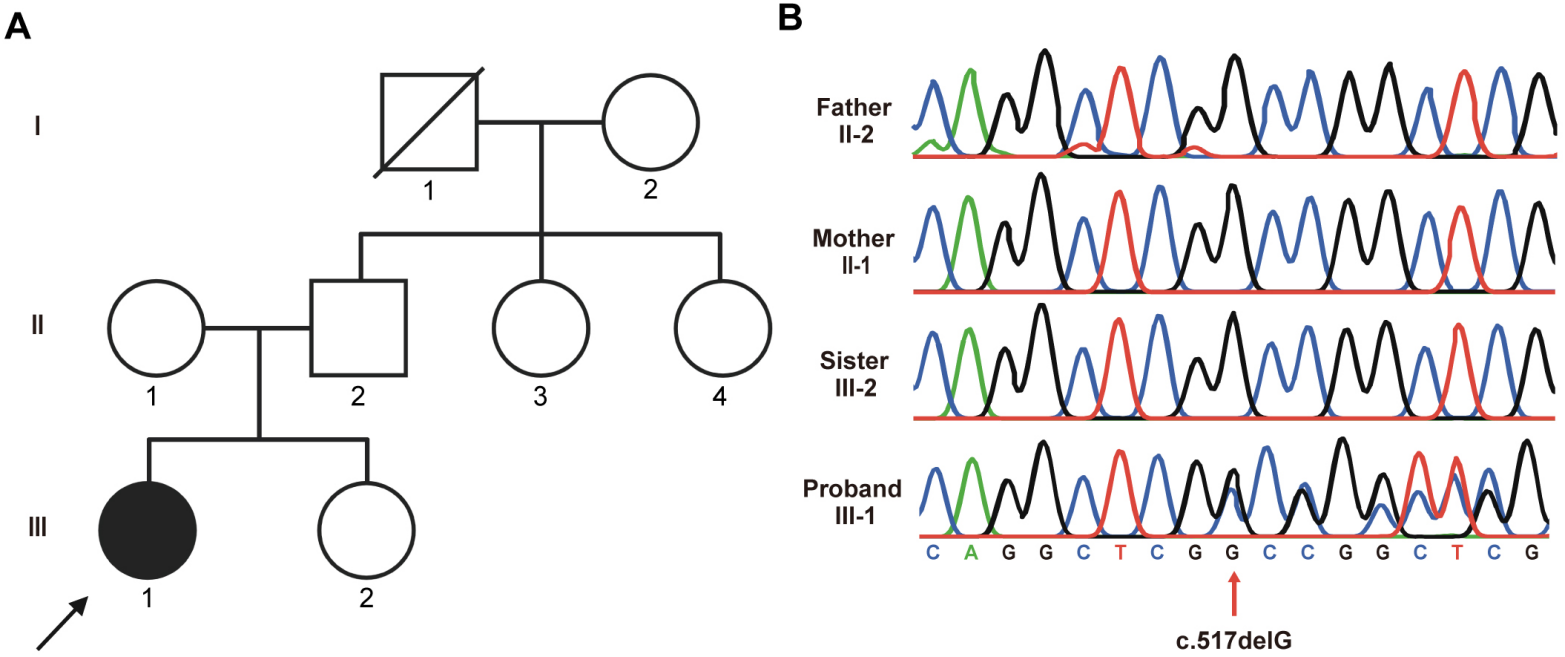
Pedigree of the patient and identification of the *GATA3* mutation via sanger sequencing. **(A)** Squares and circles represent males and females, respectively. Filled symbols indicate individuals who were affected, while open symbols indicate unaffected family members. A diagonal line through a symbol indicates a deceased individual. The arrow identifies the proband (III-1). No other family members presented with symptoms of HDR syndrome, indicating a sporadic occurrence. **(B)** Sequencing chromatograms demonstrate the heterozygous c.517delG variant in the patient. The variant is absent in the parents and the sister, indicating a *de novo* occurrence. The reference wild-type sequence is aligned at the bottom, and the arrow marks the initiation site of the frameshift mutation.

First, we analyzed the raw WES data of a 28-year-old female patient presenting with long-standing hypocalcemia and sensorineural hearing loss, but without renal abnormalities. In the Tier 1 (core parathyroid panel) analysis, we initially filtered for variants classified as ‘likely pathogenic’ or ‘not found’ in ClinVar, while restricting variant types to disruptive inframe insertions, missense variants, and splice region variants. This filtering strategy narrowed the candidate pool to four variants. Notably, a single heterozygous *de novo* frameshift variant in the *GATA3* gene (c.517delG; p.Ala173fs) was the only candidate identified with a ‘high’ functional impact score by SnpEff (**Figure 2**). Although a missense variant in *DHCR7* (c.1348C>T; p.Arg450Cys) was predicted as ‘damaging’ by SIFT and classified as ‘likely pathogenic’ in ClinVar, subsequent literature review revealed no reported association between *DHCR7* and HDR syndrome or the patient’s specific phenotype. Consequently, the *GATA3* mutation was prioritized as the primary clinical candidate. The variant introduces a premature termination codon, and the pipeline’s automated population filter confirmed its absence in the gnomAD database. This streamlined prioritization allowed for a definitive genetic diagnosis of HDR syndrome, effectively addressing the diagnostic challenge posed by the patient’s atypical clinical presentation lacking renal symptoms. The identified variant (NM_001002295.2:c.517delG) is located in exon 3 and leads to a premature termination codon at amino acid position 194 (p.Ala173fs*194). This frameshift is predicted to result in a truncated protein lacking the two highly conserved C-terminal zinc-finger domains, which are essential for DNA binding and transcriptional regulation. To validate these findings, Sanger sequencing was performed on the proband and her unaffected family members (father, mother, and sister). The presence of the c.517delG variant was confirmed only in the proband, while all family members exhibited the wild-type sequence, indicating that the mutation occurred *de novo* (**Figure 3**).

The rarity of the variant was further confirmed by cross-referencing its absence from the Exome Aggregation Consortium (ExAC) and the 1000 Genomes Project. Furthermore, this mutation is not currently registered in the ClinVar database. According to the ACMG/AMP guidelines (criteria: PVS1, PS2, PM2) (31), the variant was classified as pathogenic, causing HDR syndrome via *GATA3* haploinsufficiency.

Based on the presence of hypoparathyroidism, sensorineural deafness, and a pathogenic *GATA3* mutation identified via EVE, the patient was diagnosed with HDR syndrome despite the absence of overt renal anomalies at the time of diagnosis. Second, to validate the Tier 2 (endocrine expansion panel), we used a dataset from a patient with bilateral adrenocortical adenomas. Reflecting the patient’s clinical focus on endocrine symptoms without parathyroid involvement, the Tier 1 (core parathyroid panel) analysis correctly identified zero significant variants out of 77 candidates. Specifically, none of the 77 variants within the parathyroid panel were classified as ‘likely pathogenic’ or higher in ClinVar, nor did any exhibit a ‘high’ functional impact score via SnpEff. This result demonstrates the high specificity of the EVE pipeline in avoiding false-positive diagnoses in unrelated clinical domains. Upon extending the analysis to the Tier 2 expansion panel, which encompasses a broader set of 1,285 variants, the workflow pinpointed a missense variant in the *KCNJ5* gene (c.503T>G; p.L168R). Notably, EVE correctly re-identified the exact causative variant reported in the original study (30). Notably, among these 1,285 variants, this was the exclusive candidate that satisfied the criteria of being classified as ‘likely pathogenic’ or higher in ClinVar while simultaneously being predicted as ‘damaging’ by SIFT. No other variants in the entire expansion panel met this clinical and functional threshold. The isolation of this single, well-established somatic mutation from such a large dataset confirms the pipeline’s exceptional reliability in capturing actionable variants while effectively neutralizing genomic noise.

These validation results confirm that the EVE pipeline effectively identifies genetic variants that precisely align with documented clinical manifestations. By isolating single actionable candidates from thousands of raw variants, the workflow demonstrates high sensitivity for rare mutations and exceptional specificity against genomic noise. Ultimately, EVE transforms complex exome datasets into clear and high-fidelity clinical reports, providing a reliable foundation for rapid diagnostic decision-making in endocrine practice.

### 3.3 Clinical characteristics of the patient with the EVE-identified *GATA3* variant

**Figure 4:**
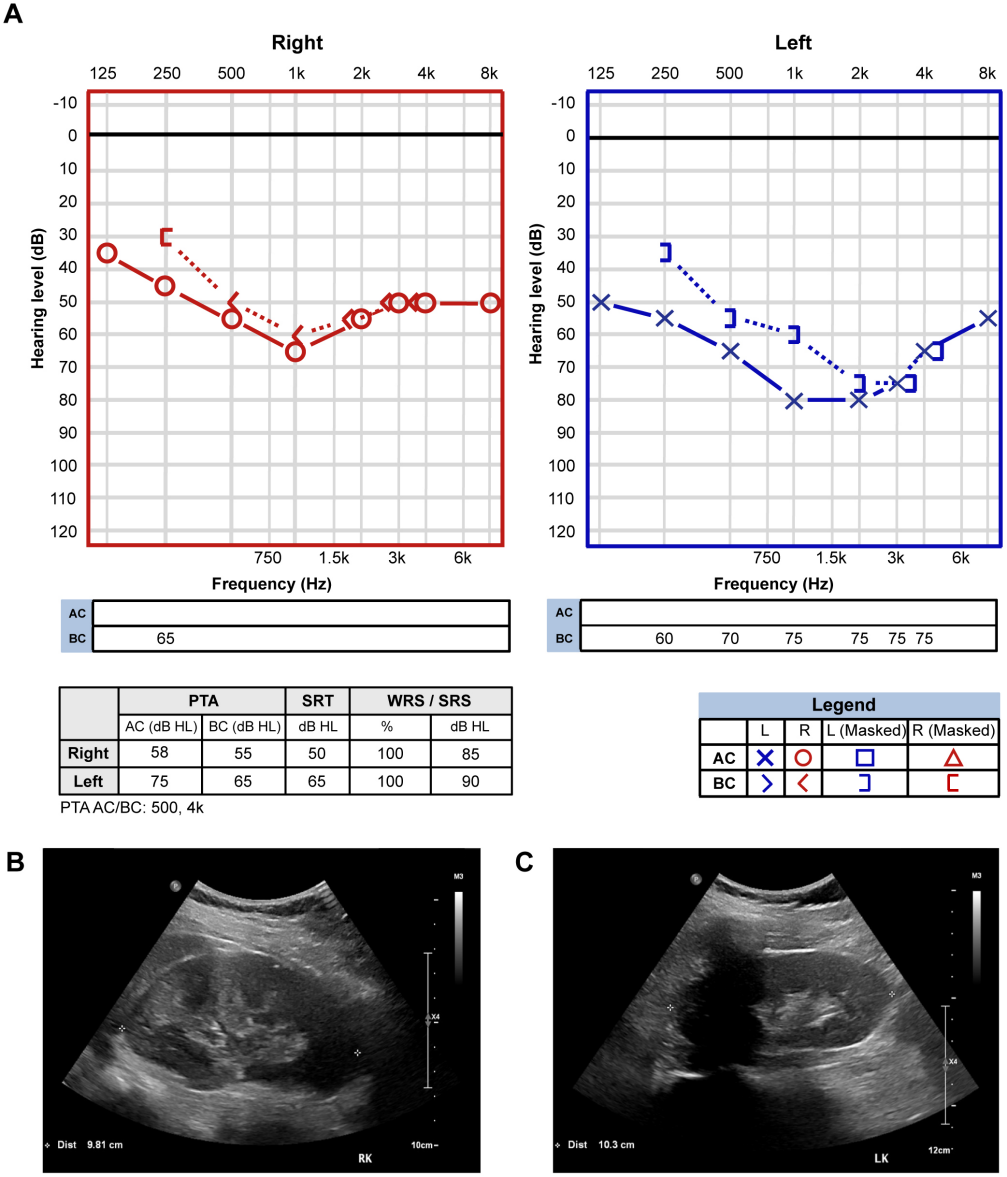
Clinical evaluation of sensorineural hearing loss and renal morphology **(A)** Pure-tone audiograms of the right (red symbols) and left (blue symbols) ears demonstrating air-conduction and bone-conduction thresholds plotted across tested frequencies. The right ear shows air-conduction thresholds consistent with moderately severe hearing loss, with a pure-tone average (PTA) of 58 dB HL. The left ear demonstrates higher air-conduction thresholds across frequencies, with a PTA of 75 dB HL indicating bilateral sensorineural hearing impairment. Speech audiometry results summarized below the audiograms show that the speech reception threshold (SRT) is consistent with the pure-tone audiometry findings, and word recognition remains preserved despite the elevated thresholds. **(B, C)** Renal ultrasonography findings. Longitudinal ultrasonographic images of the right kidney [RK, **(B)**] and the left kidney [LK, **(C)**] demonstrating preserved renal contour and corticomedullary differentiation. The bipolar length of the RK and LK measure approximately 9.8 and 10.3 cm respectively. WRS, word recognition score; SRS, speech recognition score.

The clinical presentation of the 28-year-old female patient, in whom the pathogenic *GATA3* variant was identified, is detailed as follows. The patient was referred to our endocrinology clinic with recurrent episodes of hand and foot paresthesia and intermittent muscle spasms. Her symptoms had first appeared at approximately 10 years of age with occasional progression to carpopedal tetany. She also reported long-standing bilateral hearing impairment that was noted during childhood but had not been fully evaluated until adulthood. She was previously diagnosed with idiopathic hypoparathyroidism at another institution and was intermittently treated with oral calcium supplementation. There was no history of neck surgery, autoimmune disease, radiation exposure, or severe perinatal illness. Family history was unremarkable, and no relatives had hypocalcemia, hearing loss, or kidney disease. On physical examination, the Chvostek and Trousseau signs were intermittently positive. No dysmorphic features were observed. Laboratory evaluation revealed hypocalcemia (serum calcium, 8.13 mg/dL), elevated serum phosphate levels (4.60 mg/dL), and inappropriately low intact parathyroid hormone concentrations (6.4 pg/mL) consistent with hypoparathyroidism. Serum magnesium (2.0 mg/dL) and 25-hydroxyvitamin D levels (23.7 ng/mL) were within the normal ranges. Audiological evaluations, including pure-tone and speech audiometry, demonstrated bilateral sensorineural hearing loss (**Figure 4A**). The air conduction pure-tone average (PTA), calculated using the six-division method $\frac{\left(500\;Hz\;+(1000\;Hz\;\times \;2)\;+(2000\;Hz\;\times \;2)\;+4000\;Hz\right)}{6}$, was 58 dB HL in the right ear (moderately severe hearing loss) and 75 dB HL in the left ear (severe hearing loss).

Renal ultrasonography revealed no overt structural abnormalities, including renal hypoplasia, cysts, or hydronephrosis (**Figures 4B, C**). The estimated glomerular filtration rate and urinalysis results were within normal limits (serum creatinine, 0.64 mg/dL). Given the combination of hypoparathyroidism and sensorineural hearing loss with early onset, and a pathogenic *GATA3* mutation identified via EVE, the patient was diagnosed with HDR syndrome despite the absence of overt renal anomalies at the time of diagnosis. Following this diagnosis, she was treated with oral calcium carbonate (500 mg twice daily) and calcitriol (0.25 µg daily), resulting in stabilization of serum calcium levels and improvement of neuromuscular symptoms. Due to the potential for late-onset renal manifestations, the patient was advised to undergo periodic renal surveillance, including renal function testing and imaging. Genetic counseling was provided, emphasizing the autosomal dominant inheritance pattern and the possibility of variable expressivity.

## 4 Discussion

Integration of genomic medicine into routine endocrinology practice has historically been limited by two opposing challenges; the prohibitive cost of commercial multi-gene panels and interpretative complexity of unbiased WES. Here, we describe a streamlined and clinician-oriented pipeline that effectively bridges this gap. By filtering raw WES data via a tiered architecture of high-priority endocrine genes, clinicians can harness the diagnostic power of NGS without requiring advanced bioinformatic expertise. A primary advantage of this WES-based approach over traditional targeted panels is its potential for retrospective analysis. Physical gene panels are static, whereas WES data is future-proof because the original data can be re-analyzed using our pipeline by updating the gene list and avoids the need for costly re-sequencing (32). Furthermore, by restricting the initial analysis to well-characterized genes representing the most frequent etiologies of parathyroid and broader endocrine disorders, we considerably reduce the noise of incidental findings. This focused approach addresses the practical difficulty of filtering through thousands of non-relevant SNPs (5).

In this context, we deliberately bypassed downstream statistical filtering, such as GATK’s variant quality score recalibration (VQSR) and standard hard filtering. Since our targeted virtual panel approach inherently minimizes the false discovery rate by restricting the search space to high-confidence gene regions, maintaining maximum sensitivity for rare pathogenic variants was prioritized over aggressive statistical pruning, which often requires much larger cohorts to be effective. Notably, EVE’s noise-reduction strategy enables GATK HaplotypeCaller to accurately detect both germline and somatic variants. This versatility accelerates the diagnostic process and reduces the time to personalized care, allowing clinicians to rapidly identify actionable variants without getting lost in the full complexity of the exome.

The clinical utility of this framework is demonstrated by our identification of a mutation in *GATA3* in a young woman presenting with hypoparathyroidism and sensorineural hearing loss. *GATA3* encodes a dual zinc-finger transcription factor containing N-terminal (ZnF1) and C-terminal (ZnF2) domains, encoded by exons 4 and 5, respectively. ZnF2 is critical for DNA binding, whereas ZnF1 stabilizes this binding and interacts with cofactors, such as Friends of GATA (FOG) (33). Mutations that disrupt these domains lead to haploinsufficiency, the primary molecular mechanism underlying HDR syndrome (14, 34). The frameshift variant identified in the patient (c.517delG) introduced a premature termination codon in exon 3. This alteration is predicted to disrupt *GATA3* function either through nonsense-mediated decay (NMD), leading to transcript degradation, or by generating a truncated protein lacking the critical zinc-finger domains. Notably, this case highlights significant phenotypic variability. To our knowledge, this is the first report of the c.517delG variant in Korea and the second worldwide, following an initial report in a Japanese family (35). In a Japanese study, distinct intrafamilial variability was noted; unilateral renal agenesis was observed in the female proband, whereas her affected male sibling exhibited normal renal structures. This gender-based discrepancy in the Japanese family initially suggested the possibility of sex-influenced penetrance. However, our patient, who was also a young female of similar age to the Japanese proband, presented without any renal anomalies. This phenotypic discrepancy between individuals of the same sex and genotype strongly suggests the involvement of other modifying factors, such as epigenetic mechanisms and oligogenic inheritance in determining the renal phenotype of HDR syndrome. Documenting this incomplete phenotype expands the mutational and clinical spectrum of *GATA3* and reinforces the evidence that identical variants are associated with highly variable phenotypic expression. Under such circumstances, the rapid mutation screening facilitated by EVE becomes an essential diagnostic bridge, transforming phenotypic ambiguity into molecular certainty for timely clinical intervention.

Despite these clinical successes, it is important to acknowledge the inherent limitations of this protocol. WES primarily targets protein-coding regions and may miss deep intronic variants or regulatory elements that could contribute to disease. Although our pipeline efficiently detects single nucleotide variants (SNVs) and small indels, detecting large copy number variations (CNVs), such as large deletions in *STK11* or *RET*, often requires specialized algorithms or supplementary multiplex ligation-dependent probe amplification (MLPA) testing (36). In the current implementation, the canonical transcript is defined as the longest CDS via SnpEff’s -canon option. While this approach ensures computational efficiency and maximizes exon coverage to minimize the risk of missing functional variants, the longest CDS does not always coincide with the clinically dominant transcript. To address this limitation and further enhance clinical and biological accuracy, future updates of EVE will incorporate the Matched Annotation from NCBI and EMBL-EBI (MANE) Select-based canonical transcript definition, which can be readily integrated into the existing pipeline architecture (37). Furthermore, even when pathogenic variants fall within the detectable spectrum of WES, interpretation remains a significant bottleneck. Many variants identified in practice are classified as variants of uncertain significance (VUS), providing clinicians with ambiguous results. This highlights the critical need for a community-driven endocrine variant atlas. We aim to encourage endocrinologists to report and pool variant data by standardizing the analysis pipeline. As more phenotype-genotype correlations are documented, many VUSs can be reclassified as benign or pathogenic to improve diagnostic yield globally. Including pathogenicity prediction tools such as SIFT and PolyPhen-2, along with ClinVar integration, serves as a first-line filter to aid in this stratification. To further facilitate the resolution of VUSs, future iterations of EVE will aim to integrate comprehensive population-specific and global variant databases such as KAVIAR (Known VARiants) alongside regional interpretation guidelines (38). By leveraging expansive allele frequency datasets, an automated ACMG/AMP classification module could effectively filter out high-frequency VUSs as likely benign polymorphisms, thereby narrowing the search space and accelerating the identification of true actionable variants (39). The current EVE output already includes the core evidence categories required for ACMG/AMP classification, namely functional impact (SnpEff), in silico pathogenicity predictions (SIFT, PolyPhen-2), population allele frequency (gnomAD), and clinical significance (ClinVar). The planned extension will systematically combine these annotations to assign provisional ACMG/AMP categories directly within the clinical report, further reducing the manual interpretation burden on clinicians and bringing EVE closer to a fully integrated diagnostic platform.

## Data Availability

The original contributions presented in the study are included in the article/**Supplementary Material**. Further inquiries can be directed to the corresponding authors.
