# Supplementary material for: Establishment of the endocrine variant extractor and its clinical application in identifying a novel GATA3 mutation in HDR syndrome
Source: Front Endocrinol (Lausanne). 2026 Jul 1;17:1840651. doi: 10.3389/fendo.2026.1840651 (PMC13368677; doi:10.3389/fendo.2026.1840651)
Supplement: Supplementary file 2 [file Table2.docx]

**Supplemental Table S3. List of primers**

| Name | Sequence | Description |
| --- | --- | --- |
| GATA3-sequencing-F | CTACATTTGATGGGACATCCCTGTG | PCR amplification |
| GATA3-sequencing-R | GTTTTAAATCAGATCCCACTTCCCC | PCR amplification |
